# Over-Wintering Tadpoles of *Mixophyes fasciolatus* Act as Reservoir Host for *Batrachochytrium dendrobatidis*

**DOI:** 10.1371/journal.pone.0092499

**Published:** 2014-03-19

**Authors:** Edward J. Narayan, Clara Graham, Hamish McCallum, Jean-Marc Hero

**Affiliations:** 1 Environmental Futures Research Institute, School of Environment, Griffith University, Gold Coast Campus, Queensland, Australia; 2 Environmental Futures Research Institute, School of Environment, Griffith University, Nathan Campus, Queensland, Australia; University of California-Riverside, United States of America

## Abstract

*Batrachochytrium dendrobatidis* (*Bd*), a cutaneous amphibian fungus that causes the lethal disease chytridiomycosis, has been implicated as a cause of many amphibian declines. *Bd* can tolerate low temperatures with an optimum thermal range from 17–24°C. It has been shown that *Bd* infection may result in species extinction, avoiding the transmission threshold presented by density dependent transmission theory. Prevalence of *Bd* during autumn and winter has been shown to be as low as 0% in some species. It is currently unclear how *Bd* persists in field conditions and what processes result in carry-over between seasons. It has been hypothesised that overwintering tadpoles may host *Bd* between breeding seasons. The Great Barred Frog (*Mixophyes fasciolatus*) is a common, stable and widespread species in Queensland, Australia, and is known to carry *Bd*. Investigation into *Bd* infection of different life stages of *M. fasciolatus* during seasonally low prevalence may potentially reveal persistence and carry-over methods between seasons. Metamorphs, juveniles, and adults were swabbed for *Bd* infection over three months (between March and May, 2011) at 5 sites of varying altitude (66 m–790 m). A total of 93 swabs were analysed using Polymerase Chain Reaction (PCR) real-time analysis. PCR analysis showed 6 positive (1 excluded), 4 equivocal and 83 negative results for infection with *Bd*. Equivocal results were assumed to be negative using the precautionary principle. The 5 positive results consisted of 4 emerging (Gosner stage 43–45) metamorphs and 1 adult *M. fasciolatus*. Fisher's exact test on prevalence showed that the prevalence was significantly different between life stages. All positive results were sampled at high altitudes (790 m); however prevalence was not significantly different between altitudes. Infection of emerging metamorphs suggests that individuals were infected as tadpoles. We hypothesise that *M. fasciolatus* tadpoles carry *Bd* through seasons. Thus, *Mixophyes fasciolatus* may act as disease reservoirs at multiple life stages.

## Introduction

Amphibian species world-wide have experienced rapid population declines and extinction in the recent decades. The extinction rate between 1980 and 2004 may be up to 200 times greater than the historical rates of previous centuries [1]. Many of these declines and extinctions have occurred in undisturbed and protected habitats [2], [3], [4]. In these cases the anthropogenic means usually cited for species extinctions—including habitat disturbances, fragmentation and the introduction of exotic pests and predators—fail to satisfactorily explain the level of declines observed [2], [5]. As a result declines in protected areas have been labelled enigmatic [2].

Chytridiomycosis, an epidermal disease of amphibians caused by the fungal pathogen *Batrachochytrium dendrobatidis* (hereafter *Bd*), has been cited as the cause of many enigmatic declines (for example [6], [7]). Infecting the keratinised epidermis of amphibians, *Bd* causes death in adult amphibians by disrupting cutaneous osmoregulation, which causes electrolyte and osmotic imbalances leading to cardiac failure and death [8]. The symptoms of lethal chytridiomycosis include: loss of the righting reflex; thickening and lesions of the superficial epidermis; and enlargement, degeneration and vacuolation of cells within the superficial epidermis [9], [10]. Despite the lethal nature of chytridiomycosis, infection with *Bd* does not always result in mortality. Species and populations of species show differing levels of susceptibility and some species persist without obvious declines when infected with *Bd* [11], [12].

Declines in amphibians have occurred at a higher rate at high altitudes and latitudes [13], [14] and 70% of Australia's threatened amphibian fauna occur at high altitudes [15]. These patterns of declines have been linked to niche occupation and rainforest specialists; in addition species whose life histories are closely associated with permanent streams are at a higher risk of decline [15], [16], [17], [18]. High altitude declines have also been hypothesised to be due to the cool moist conditions of high altitudes increasing disease virulence [19]. *Bd* has an optimum temperature range between 17 and 24 °C and cannot survive desiccation or prolonged exposure to temperatures above 28 °C [20], [21], [22].

The ability of a disease to drive a species to extinction depends on transmission methods and availability of a reservoir host. Classical density dependent transmission theory states that transmission increases with density and therefore as a disease drives declines, density reaches a point at which transmission rates are too low to cause extinction [23], [24]. To avoid this transmission threshold and drive species extinction, the disease must have a reservoir host [24]. The diverse range of amphibian species infected by *Bd* may provide a transmission reservoir and allow *Bd* to persist in more resistant populations while driving vulnerable populations to extinction [25]. The Bullfrog *Rana catesbeiana* is a good example of a reservoir host and due to the amphibian trade has introduced populations globally, many of which are *Bd* positive [25], [26], [27]. Although testing positive for *Bd, R. catesbeiana* shows no symptoms of the lethal disease chytridiomycosis [25]. In Australia, the widespread Stony Creek Frog (*Litoria wilcoxii*), infected with *Bd* but not experiencing decline, has also been implicated as a disease reservoir [12], [28].

Life stages play an important role in the disease dynamics of *Bd,* as infections in tadpoles only occur on the keratinised mouthparts and are mostly sub lethal [29], [30]. [31] have recently hypothesised that tadpoles are a possible reservoir for *Bd*, representing a less vulnerable life stage that does not succumb to the disease, with an aquatic life history making them vulnerable to infection. Many amphibian species have prolonged larval stages with larvae overwintering in permanent water bodies. [31] have shown the successful laboratory transmission of *Bd* to tadpoles and between tadpoles, and this was further validated in the field by [24].

*Bd* has been shown to have a seasonal pattern in some species of Australian frogs, with high prevalence in winter and spring and low prevalence in autumn and summer [12], [28]. [12] have shown that *Bd* prevalence in *Litoria wilcoxii* may be as low as 0% in autumn months, and it is currently unclear how *Bd* prevalence is sustained through seasons.

In this study, we hypothesise that overwintering tadpoles provide a good reservoir for *Bd* between seasons and during lethal epidemic outbreaks. We also hypothesise that altitude is an important factor in this relationship. Therefore, we investigated: (1) the prevalence of *Bd* in the Great barred frog (*Mixophyes fasciolatus*) in autumn; (2) the patterns of *Bd* prevalence between life stages, in metamorphs, juveniles and adults; and (3) the patterns of *Bd* prevalence between high and low altitudes.

## Methods

### 2.1 Ethics statement

The Queensland Government Department of Environment and Research Management (QLD DERM) issue permit for the research to be conducted (Permit # WITK07104610). Furthermore, the Animal Ethics Committee of Griffith University, Australia issues permit for this research (permit number/ENV/08/10/ENV). Field studies did not involve endangered or protected species. No animals were scarified during this study. Frogs were captured manually using hand-capture by wearing non-powdered sterile hand gloves.

### 2.2 Study Species

The species targeted for this study was *Mixophyes fasciolatus,* a common and widely distributed frog, inhabiting the banks of montane creeks, streams, and isolated ponds and dams. Although found in a wide range of habitat types from dry sclerophyll to rainforest, *M. fasciolatus* shows an affinity for wetter habitats. Its distribution ranges from the Clarke Range on the mid-South East Queensland (SEQ) Coast to Dorrigo on the Northern New South Wales (NSW) East Coast. The breeding season of *M. fasciolatus* occurs from November to March during spring and summer (Parris 2002). *M. fasciolatus* is known to breed in both lentic and lotic permanent water bodies. Eggs are laid in the leaf litter and on logs and trees, to be washed into the water during rain [32], [33]. Tadpoles have been observed overwintering [32] and have been known to take up to twelve months to metamorphose [30]. The population status of *M. fasciolatus* is stable. Nevertheless *Bd* has been found in *M. faciolatus* populations in central and south east Queensland including: Kroombit Forest Reserve; Conondale National Park; Mount Mee Forest Reserve; Main Range National Park; Delicia Road Conservation Park; Miala National Park; Mount Glorious; Eungella National Park; Bunya Mountains National Park and Lamington National Park [34]. Other species of the *Mixophyes* genus have experienced recent declines and the Fleay's barred frog (*M. fleayi*) and the Giant barred frog (*M. iteratus*), are currently listed as endangered in NSW and QLD under the *NSW Threatened Species Conservation Act 1995 (NSW)* and the *Nature Conservation (Wildlife) Regulation 2006 (QLD)* respectively. It has been hypothesised that the wide habitat utilization of *M. fasciolatus* has protected it from population decline [32]. *M. fasciolatus* was chosen for this study due to its widespread distribution at all elevations in SEQ, stable population status and because *Bd* is known to infect the species it is expected to be a disease reservoir.

### 2.3 Field Sampling

**Figure 1:**
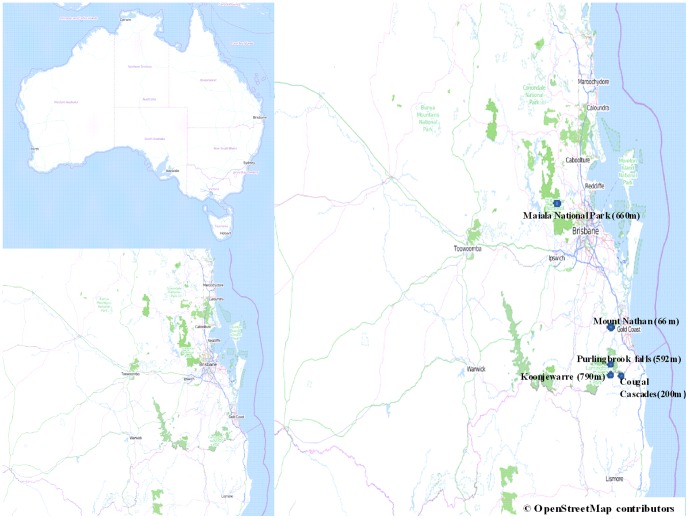
Map of study sites including two low and three high altitude sites in south east Queensland, Australia. Map was plotted using OpenStreetMap contributors.

**Figure 2:**
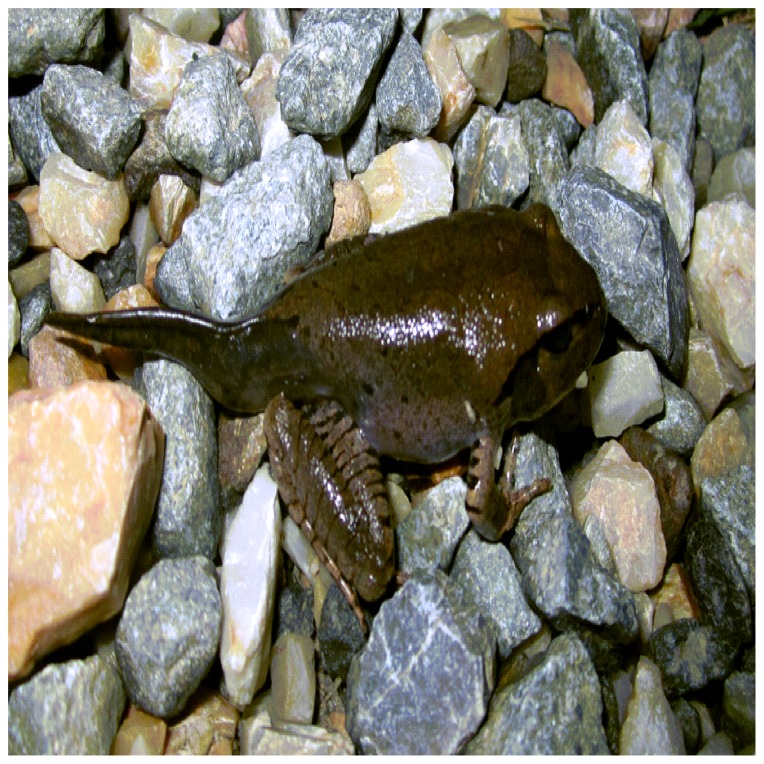
*Mixophyes fasciolatus* metamorph Gosner stages 43–45, sampled as emerging from water. (Photo: Clara Graham).

Sampling occurred from the beginning of March 2011 to the end of May 2011. Sampling took place at five sites consisting of two low altitude and three high altitude sites in SEQ, Australia (Fig.1). The low altitude sites were located at Mt Nathan (27°58′36.77″S, 153°16′36.09″E) in Numinbah Valley at an elevation of 66 m, and at Cougal Cascades (28°14′11.64″S 153°20′51.19″E) in Currumbin National Park (NP) at an elevation of 200 m. The three high altitude sites were located at Koonjewarre (28°13′40.58″S 153°16′19.26″E) and Purling Brook Falls (28°11′19.20″S 153°16′10.22″E) in Numinbah Valley, Lamington NP and Maiala NP Picnic Grounds (27°19′45.66″S 152°45′46.71″E) in Mt Glorious with altitudes of 790 m, 592 m and 660 m respectively. Sites with an altitude greater than 400 m were considered high altitude sites, while those less than 400 m were low altitude sites. All sampling locations contained a permanent water body within the sampling area. *Bd* intensity was measured using the protocol of [35] by swabbing the skin of the frog a total of 35 times, five times on: each side; the ventral surface; the inner thigh of the hind legs; and both hind feet [36]. Samples were taken using Medical Wire MW 100–100 swabs. A total of 93 swabs were taken: 52 from adults; 17 from juveniles (Gosner stage 46); and 24 from late stage metamorphic (Gosner stages 43–45, Fig. 2) *M. fasciolatus* [37].

### 2.4 Laboratory analysis

A zoospore suspension of *B. dendrobatidis* isolate AAHL (Australian Animal Health Laboratory) 98 1810/3 was used to prepare standards. PCR analysis was done at the EcoGene Laboratory, Landcare Research Centre, New Zealand. Real- time Taqman Polymerase Chain Reaction (PCR) techniques of [38] which targeted the intergenic transcribed spacer 1 (ITS1) region specific to *B. dendrobatidis* were used to analyse swabs. As discussed by [39] inferring infection intensity from amplification of variable copy loci such as the ITS can be a limitation with this PCR technique (see discussion 2.4.1 with EcoGene Laboratory technician - Julia Allwood). Furthermore, the authors [39] recommended that single ITS1 PCR-amplicons should be used as the absolute standard in order to reduce error in estimates of *Bd* zoospore loads from field studies.

#### 2.4.1 Key limitations of inferring infection intensity from amplification of variable copy loci such as the ITS

There are several key issues with using a variable copy marker to infer infection intensity. In brief, typically standards of zoospore equivalents are used in comparison to sample swab chytrid ‘load’ (after accounting for dilution factors etc.). These standards are originally generated via visual counting of spores, extraction etc. This act in the first place relies on the key assumption that the standards are truly reflective of zoospore genome quantity, which is questionable due to variable copy number to begin with, and that samples are not being inhibited in some way. The results are then used to estimate the zoospore quantity within each sample. The real-time PCR method we employed currently tries to circumvent this by offering zoospore estimates. However, this evaluation makes no inference as to how this estimate relates to infection levels. Furthermore, inferences of infection intensity in relation to spore counts are debatable, regardless of ITS copy number. This is because a lot of the original work for developing a real-time quantitative PCR (now widely used) capable of zoospore estimation was conducted on captive populations it is now deemed unlikely that the acceleration of the infection in these studies is truly reflective of infected frogs in the wild. Therefore, data sets on animal health and environmental factors are necessary to make conclusions with regards to the effects of infection estimates on the health of the amphibian. Comparative assessment of physiological stress through baseline glucocorticosteroid levels would be helpful to make conclusions [40], [41]. Finally, the use of a genetic marker that is found in variable copies is not recommended for strictly quantitative investigations. However, even if putting forward an estimate then perhaps inferring the range or ball park above and below that would help, although that too depends on what level of copy number variation is seen in that locus (i.e. 1–5 copies is a lot easier to interpret that 10–20 copies). In saying that, variable copy number loci are useful as presence/absence markers for samples of low DNA quantity, and often quality, as the multiplication of the target increases the odds of at least one copy successfully amplifying. Using a conventional PCR would be better suited for applied health studies this is more cost effective in both reagents and shelf lives etc., but also because it has been shown to be as sensitive as the real-time method when applied correctly.

The Sample size at lowland sites was low but adequate (N = 19) and this was made up of metamorphs (N = 1), Juveniles (N = 1) and adults (N = 17). Sample size at highland sites were larger (N = 74), with 16 juveniles, 23 metamorphs and 35 adults. The air temperature, cloud cover and precipitation was observed on each sampling occasion and all frogs had their mass weighed in grams and their snout vent length (SVL) measured in centimetres. After all measurements were taken frogs were uniquely toe-clipped using the methods of [42], to avoid sampling bias through recapture of the same individual.

### 2.5 Statistical Analysis

The statistical computation software R (R Development Core Team 2011) was used to perform Fishers exact tests and calculate exact 95% binomial confidence intervals.

## Results

Real time PCR analysis of swabs returned 6 positive, 4 equivocal (when run in triplicates, at least one of the three wells are negative) and 83 negative results for infection with *Bd*. The identity of 1 positive result was unable to be obtained due to labelling errors, and was excluded from further analysis. We also highlight caution in interpreting these results as sample sizes were low. The remaining 5 positive results consisted of 4 metamorphic and 1 adult *M. fasciolatus.* The 4 equivocal results consisted of 1 adult and 3 metamorphs; however observing the precautionary principle, all equivocal results are being treated as false positives and were also excluded from analyses.

For *Bd* infection to lead to declines, the number of zoospores present on an individual needs to be greater than approximately ten thousand zoospore equivalents [43]. Intensity of *Bd* spores on positive individuals was low, with a range between 0.74 and 5.2 zoospore equivalents; one metamorph had a slightly higher zoospore equivalent of 73.

**Figure 3:**
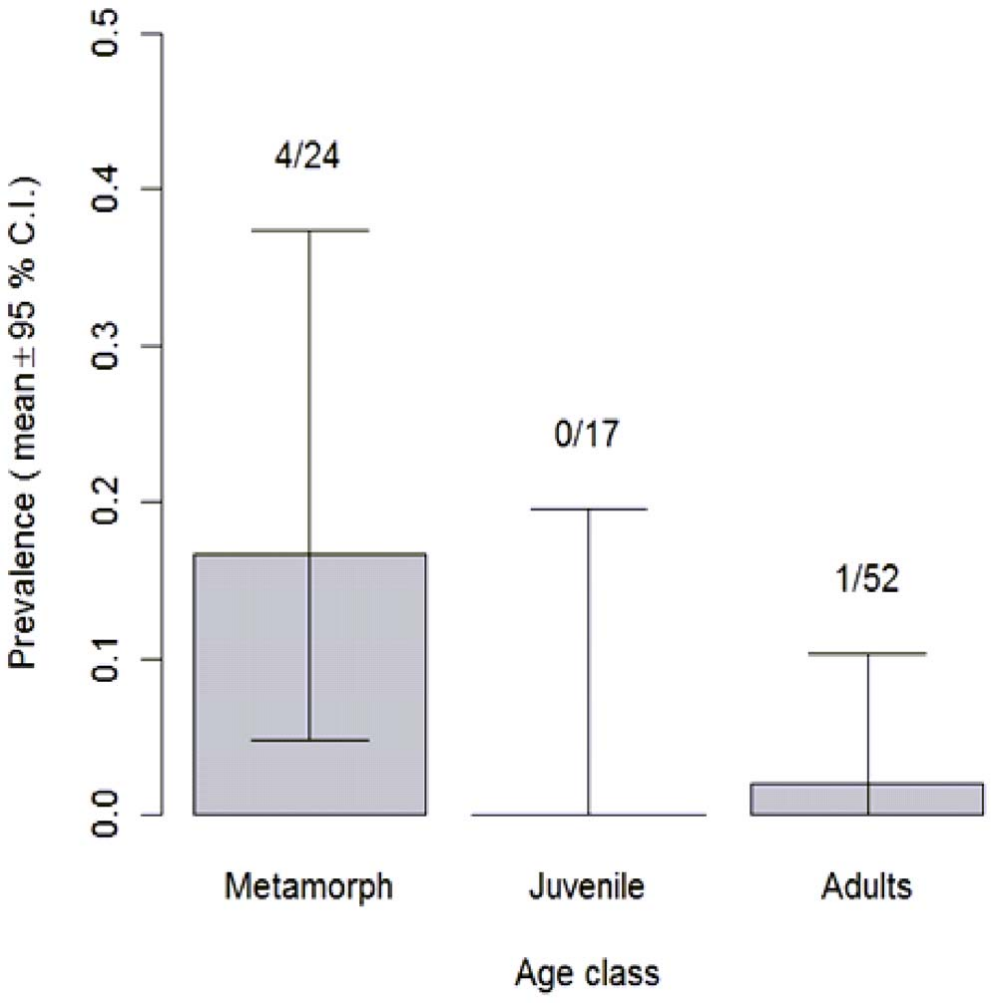
The distribution of *Bd* prevalence (positive/total) between life stages. Exact binomial confidence intervals for the estimated prevalence in the population for each life stage are shown. Prevalence differs significantly between life stages (Fishers exact test, P = 0.027<0.05).

The overall prevalence of *Bd* in *M. fasciolatus* was low at 6.5% (infected  = 6, N = 93). Prevalence was significantly different between life stages (Fisher's exact test, P = 0.027 <0.05) (Fig. 3) and was highest in metamorphs at 16.6% (infected  = 4, N = 24).

**Figure 4:**
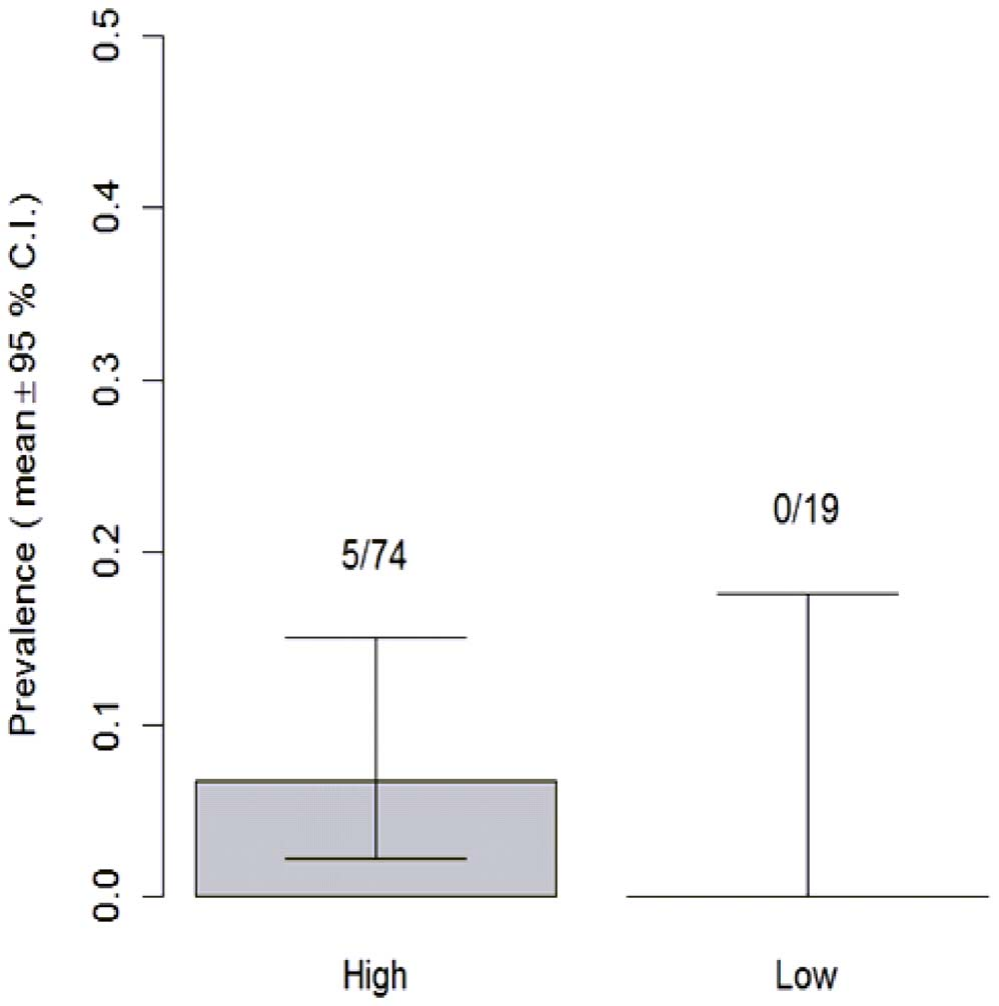
The distribution of *Bd* prevalence (positive/total) between altitudes. Exact binomial confidence intervals for the estimated population prevalence at each altitude are shown. No positive results were found at low altitudes. Prevalence in high altitude populations was estimated at 0.054. The difference between altitudes is not significant (Fisher's exact test, P = 0.58>0.05).

The prevalence of *Bd* at high altitudes was 5.4% (infected = 5, N = 74), while prevalence at low altitudes was 0% (infected  = 0, N = 19). Fishers exact test showed that the differences between altitudes were not significant (P = 0.58>0.05) despite no positive results being found at low altitudes (Fig. 4). The lack of significant difference between altitudes is most likely a result of low sample size, particularly at low altitudes, and low prevalence and intensity making it difficult to pick up any patterns present.

All positive results were sampled at Koonjewarre (28°13′40.58″S 153°16′19.26″E), a high altitude site on the 15^th^ and 21^st^ of March, 2011. The prevalence of *Bd* at Koonjewarre was 9.8% (infected = 5, N = 51). Sampling at this site occurred from 15^th^ to the 24^th^ of March. The air temperature at the site on the 15^th^ and 21st was 18.7 and 21°C respectively. The closest weather station, The Gold Coast Seaway located at sea level recorded a mean minimum of 21.1 °C and a mean maximum of 28.5 °C for the month of March, 2011.

## Discussion

During autumn, *Bd* is present in *M. fasciolatus* in SEQ with low prevalence and intensity in adults and emerging metamorphs. Metamorphs were the most likely life stage to be infected. Positive results were found at one highland site only but the effect of altitude was not statistically significant. The observed overall prevalence (6.5%, infected  = 6, N = 93) confirms the results of previous studies on autumn prevalence in SEQ, which have shown low autumn *Bd* prevalence which has been recorded as low as 0% in *Litoria wilcoxii* [28], [44].

At an altitude of 790 m, the site at which *Bd* was detected was 130–198 m higher than the other high altitude sites. Higher elevation may result in lower daily temperature maxima and differences in precipitation patterns, which may explain differences in *Bd* prevalence between high altitude sites. *Bd* does not survive desiccation or temperatures above 28 °C and it is possible that daily maxima at other high altitude and lowland sites prevented continued autumn prevalence. Another possible explanation for the observed null prevalence at lowland and other high altitude sites is that prevalence was too low for our sampling power to pick up. The estimated confidence intervals of the lowland samples show a large interval of variation due to the low sampling size (N = 19). A larger sampling size at lower altitudes would reduce this variation and help reveal any underlying patterns.

Newly emerged metamorphs were the most commonly infected life stage observed in this study, accounting for 4 out of the 5 infected individuals. The positive *Bd* status of newly emerging metamorphs (Gosner stage 43–45) strongly suggests that infection occurred as tadpoles. It has been shown that tadpoles can be infected with *Bd* through inoculation in the laboratory and by contaminated water in field conditions [24], [31]. If *M. fasciolatus* tadpoles are infected with *Bd* (as this study strongly suggests) and they overwinter, *M. fasciolatus* tadpoles may be acting as a disease reservoir for carryover between seasons. This hypothesis is supported by the results of [24] that implicated *Rana mucosa* tadpoles as a reservoir host and showed that they overwinter and carry *Bd* in temperatures as low as 4°C. *Mixophyes fasciolatus* are known to overwinter and can take up to 12 months to metamorphose. [30] has previously shown that *M. fasciolatus* tadpoles do carry *Bd* and this study has extended this knowledge to show that they continue to carry *Bd* into metamorphosis.

*Mixophyes fasciolatus* shows potential to be a reservoir host, as it is not experiencing any observed declines, can be infected with *Bd,* and has the potential to infect other individuals with *Bd* at multiple life stages. Low prevalence and low intensity of infection suggest that in autumn the force of infection onto other species arising from infections in *M. fasciolatus* would be low, but this may not be the case for all seasons. [31] have shown that transmission between larvae of different species can occur and [45] has shown that susceptibility to lethal infection differs between larval species. The ability of *M. fasciolatus* larvae to overwinter provides a carryover reservoir for *Bd* between seasons in a host that is tolerant of infection at both the larval and adult life stages. These characteristics provide *M. fasciolatus* with the ability to continue the cycle of *Bd* infection from the aquatic environment as larvae, to the terrestrial environment as adults and back to the aquatic environment during the subsequent breeding season.

Maintenance of *Bd* prevalence between seasons is an important factor in disease dynamics. In eastern subtropical Australia *Bd* prevalence is typically high in spring and winter and low in autumn and summer [12], [28], [44]. Frogs are most at risk of succumbing to lethal chytridiomycosis as metamorphs [46] and the potential for *M.fasciolatus* to pass *Bd* to more susceptible species at this life stage warrants further investigation.

Recently, it has been shown by [47] that tadpoles of *Rana catesbeiana*, a species commonly implicated as a reservoir host in the Americas, have the ability to mount an immune response to infection with *Bd*. Circulating white blood cell counts and neutrophils increased while eosinophil decreased in response to *Bd* infection [47]. Other species with a tolerance to *Bd* may have a similar response to infection with *Bd* and the immune response of *M.fasciolatus* at different life stages is worthy of investigation. Furthermore, most recently, we have also shown that adult *M. fasciolatus* have much higher stress hormone (corticosterone) levels than their lowland counterparts in the absence of *Bd* prevalence [40]. We have also shown in *Litoria wilcoxii* that individuals with *Bd* positive prevalence have much higher levels of baseline corticosterone in comparison to *Bd* negative prevalence individuals [41]. Whether or not *Bd* causes sub-lethal stress in over-wintering tadpoles warrants urgent investigation. Furthermore, the use of conservation physiology tools for assessing the sub-lethal impacts of *Bd* on amphibian fitness is highly recommended [48].

In conclusion, this study confirmed and extended the previous results of [30] to show that *M. fasciolatus* are *Bd* positive as larvae and post metamorphosis. These results strongly implicate *M. fasciolatus* as a disease reservoir for *Bd* between seasons and species. *Mixophyes fleayi* a sympatric endangered species under the *Nature Conservation (Wildlife) Regulation 2006 (QLD)* was observed at one site (Mt Glorious) were *M. faciolatus* was sampled in this study and the persistence of *Bd* in *M. fasciolatus* may be an important factor in *M. fleayi* declines. Further investigation of this pattern using a larger sampling power is important and necessary to expose the significance of the relationships observed in this study. Furthermore, investigation into the stress physiology and immune defences of *M. fasciolatus* may shed light on the sub-lethal effects on eco-physiological traits of non-declining *Bd* infected species. The occurrence of endangered species such as *M. iteratus* and *M. fleayi* in the distributional range of *M. fasciolatus* make understanding the role of *M. fasciolatus* as a disease reservoir important to management decisions.
